# Photophysical and Electroluminescence Characteristics of Polyfluorene Derivatives with Triphenylamine

**DOI:** 10.3390/polym11050840

**Published:** 2019-05-09

**Authors:** Qiang Zhang, Po-I. Wang, Guang Liang Ong, Shen Hoong Tan, Zhong Wei Tan, Yew Han Hii, Yee Lin Wong, Khee Sang Cheah, Seong Ling Yap, Teng Sian Ong, Teck Yong Tou, Chen Hon Nee, Der Jang Liaw, Seong Shan Yap

**Affiliations:** 1 Department of Chemical Engineering, National Taiwan University of Science and Technology, Taipei 10607, Taiwan; zhangyunyiyi@gmail.com (Q.Z.); wangpoi.ntust@gmail.com (P.-I.W.); 2 Faculty of Engineering, Multimedia University, Persiaran Multimedia, Cyberjaya 63100, Malaysia; ogeeal@gmail.com (G.L.O.); shenhoong5059@gmail.com (S.H.T.); zacktan93@hotmail.com (Z.W.T.); joshuahiiyewhan@gmail.com (Y.H.H.); yeelin823@gmail.com (Y.L.W.); kheesang@gmail.com (K.S.C.); seantso90@gmail.com (T.S.O.); tytou_mmu@yahoo.com (T.Y.T.); seongshan@gmail.com (S.S.Y.); 3 Department of Physics, University of Malaya, Lembah Pantai, Kuala Lumpur 50603, Malaysia; slyap@um.edu.my

**Keywords:** semiconducting polymer, electroluminescence, polyfluorene, triphenylamine, PLED, Suzuki coupling, OLED

## Abstract

In this work, polymers of poly[(9,9-dioctylfluorenyl-2,7-diyl)-co-triphenylamine] with side chains containing: pyrene (C1), diphenyl (C2), naphthalene (C3), and isopropyl (C6) structures were synthesized via a Suzuki coupling reaction. The structures were verified using NMR and cyclic voltammetry measurements provide the HOMO and LUMO of the polymers. The polymer with pyrene (C1) and naphthalene (C3) produced photoluminescence in the green while the polymer with the side chain containing diphenyl (C2) and isopropyl (C6) produce dual emission peaks of blue-green photoluminescence (PL). In order to examine the electroluminescence properties of the polymers, the solutions were spin-coated onto patterned ITO anode, dried, and subsequently coated with an Al cathode layer to form pristine single layer polymer LEDs. The results are compared to a standard PFO sample. The electroluminescence spectra resemble the PL spectra for C1 and C3. The devices of C2, C3, and C6 exhibit voltage-dependent EL. An additional red emission peak was detected for C2 and C6, resulting in spectra with peaks at 435 nm, 490 nm, and 625 nm. The effects of the side chains on the spectral characteristics of the polymer are discussed.

## 1. Introduction

Conjugated polymers are attractive because of the combined advantage of flexibility and conductivity for electronics and various forthcoming applications [1,2,3,4,5,6]. Since 1990, electroluminescence (EL) has been observed from a variety of conjugated polymers [7]. It was first shown that multiple emission colors can be obtained using blending polymers with different emission and charge-transport characteristics [8]. Polyfluorene (PF) is one of the promising polymers for both organic solar cell and light emitting diodes because of the large bandgap that enable blue fluorescence [9]. The capability makes polyfluorene the first family that emits in the whole visible spectra, from blue to red [10]. Single emission was typically obtained from each polymer, but the findings infer that multiple emissions may be achieved via modification and proper design of the polymers. The introduction of a copolymer or side chain into polyfluorene, for example, further increases the tunability of the emission spectra of the polymer, showing that multicolor emission from a single polymer component is possible [11]. Red-green-blue emission from a single polymer was achieved recently from a star-shaped polymer [12], where PF provided emission in the blue, and modification in the core produced green emission, and finally, the addition of a dopant contributed the red emission. The dopant can consist of metal complexes, dye, or quantum dots that were incorporated into the polymer [11,12,13,14]. In order to stabilize the blue emission from PF, triphenylamine (TPA) has been used with PF. A TPA-based polymer also exhibits good hole-transporting properties for organic electronics [15]. PF derivatives bearing TPA have been shown to result in a two-dimensional conjugated system that suppress π-stacking/aggregation, improves hole injection, and facilitates intramolecular energy transfer in single- and double-layer OLEDs [16]. PF-based copolymers containing TPA in the side chain improve the thermal and morphological stability and act as an antioxidant, thus stabilizing the blue emission from poly(9,9-di-n-octylfluorenyl-2,7-diyl) PFO [17]. The presence of a triphenylamine-based hydrazone comonomer with PF also improved the device performance as compared to those with a PF homopolymer [18].

In this work, we synthesized and investigated the spectral characteristics of four polymers constituting a PFO and TPA backbone of poly[(9,9-dioctylfluorenyl-2,7-diyl)-co-triphenylamine] with side chains containing either: pyrene (C1), diphenyl (C2), naphthalene (C3), or isopropyl (C6). The effects of different side chains on the photophysical and electroluminescence spectral characteristics are compared and discussed. Polymers C1 and C3 exhibited rather different emission characteristics as compared to C2 and C6 when optically excited. The difference can also be observed when the polymers are fabricated into single layer OLEDs with the structure of ITO/polymer/Al. 

## 2. Experimental 

### 2.1. Polymer Synthesis

**Figure 1:**
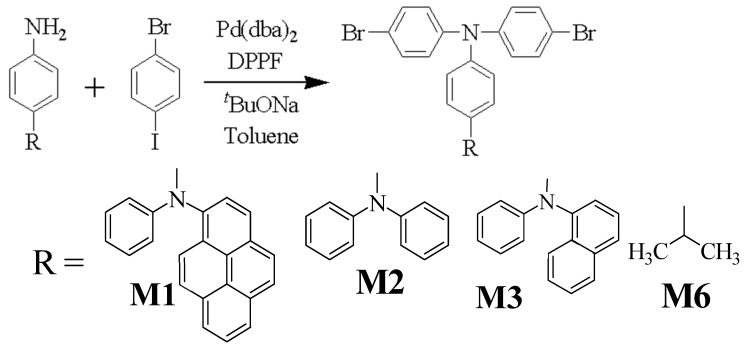
The Buchwald–Hartwig amination process was used to produce M1, M2, M3, and M6.

**Figure 2:**
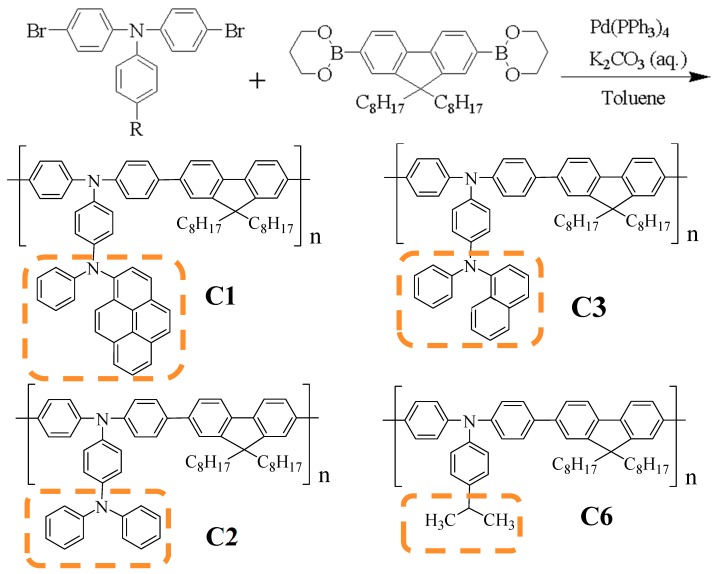
Polymers C1, C2, C3, and C6 are synthesized via the Suzuki coupling method.

The dibromo monomers of M1, M2, M3, and M6 were synthesized via Buchwald–Hartwig amination as shown in Figure 1. Suzuki coupling polymerization yielded the polymer with M1, M2, M3, and M6 in Figure 2. The synthesis processes are described in the Supplementary Information and in our previous work [15,19,20]. The details of C6 are given in the supplementary data (Figures S1 and S2), while the data for C1, C2, and C3 are derived from our previous work. The structures were verified using NMR (Figure S3) and cyclic voltammetry measurements (Figure S4) to provide the HOMO and LUMO of the polymers as shown in Table 1. 

### 2.2. OLED Fabrication

**Figure 3:**
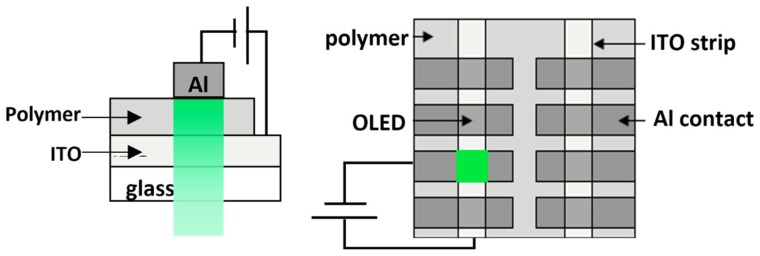
Schematic of the side view and top view of the polymer OLED device structure.

The synthesized polymers in the form of powders were first dissolved in chloroform to obtain different concentrations in a glove box. As a comparison, commercial poly(9,9-di-n-octylfluorenyl-2,7-diyl) (PFO) (Ossila, Sheffield, UK) was also used and dissolved in the same solvent. The concentrations of the polymer solution were kept at 5–6 mg/mL, except for C1, which had a lower solubility, thus a concentration of 2 mg/mL was used. In order to fabricate OLED devices with the polymers, ITO-coated glass (2.5 cm × 2.5 cm) with a resistivity of 2 × 10^−4^ Ω·cm were etched with HBr into 2 mm strips to define the anode. After wet cleaning and UV ozone cleaning for 10 min, polymer solutions were spin-cast onto the ITO substrates at 2000 rpm and annealed at 60 °C for 30 min. Subsequently, an Al layer with thickness ≈100 nm was deposited by using a thermal evaporator (Edwards Auto 306, Edwards, Burgess Hill, UK) as the cathode. The resultant thicknesses of the polymer layer of C2, C3, C6, and PFO were ≈50–80 nm, while C1 was ≈30 nm. The thicknesses of layers were measured by using a profilometer (Mahr Perthometer S2, Mahr, Göttingen, Germany). The absorbance (Abs) and photoluminescence (PL) spectra of the polymer films prepared from solution with different concentrations were measured using a UV-vis NIR spectrometer (Avantes, Apeldoorn, The Netherlands), and excited with 368 nm light to obtain the PL spectra. The single-layer device of ITO/polymer/Al is shown in Figure 3. The I–V characteristics were measured using a source measurement unit (Keithley 236) and the electroluminescence (EL) spectra were captured using a spectrometer (200–1100 nm) (Avantes AvaSpec-2048L, Avantes, Apeldoorn, The Netherlands).

## 3. Results and Discussion

### 3.1. Absorption and Photoluminescence Spectra

**Figure 4:**
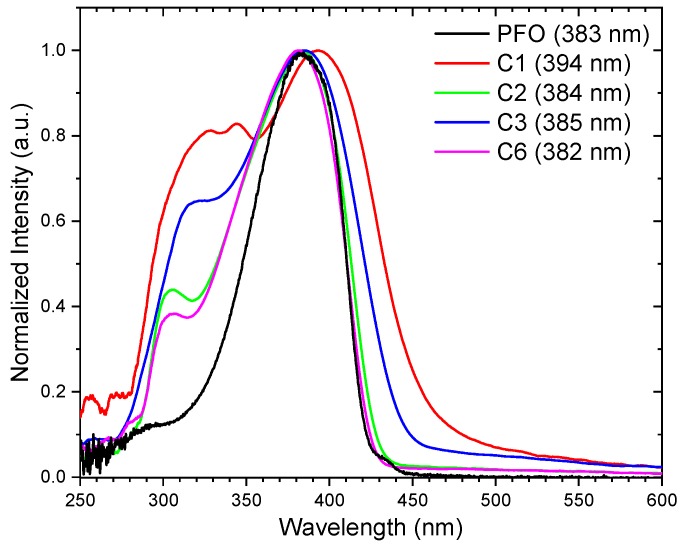
Normalized absorbance of C1, C2, C3, and C6 as compared to a PFO film (black). The maximum absorption wavelengths for each polymer are given in brackets.

The absorption spectra of all the polymer films were compared to the absorption of a commercial PFO sample in Figure 4. All the polymers absorbed from ≈300 nm to 420–450 nm depending on the polymer samples. The maximum absorption peak for PFO was obtained at ≈383 nm because of the π−π* transition. When the backbone was modified with TPA in C1, C2, C3, and C6, the absorption peaks were broadened. In addition, the main peak at 380–390 nm was red-shifted for C1 and C3. Additional peaks at 320–348 nm were detected for C1, where it was reported to be due to the pyrene structure in the side chain [21]. C3 with a naphthalene structure in the side chain had a lower peak at ≈310 nm. The maximum absorption peaks of C2 and C6 were close to that of a PFO. Another peak was detected at ≈300 nm, which has been reported for the triphenylamine unit in the polymer [15,22]. It was clearly distinguishable for C2 and C6 with a less rigid side chain, but was not well resolved for C1 and C3. 

**Figure 5:**
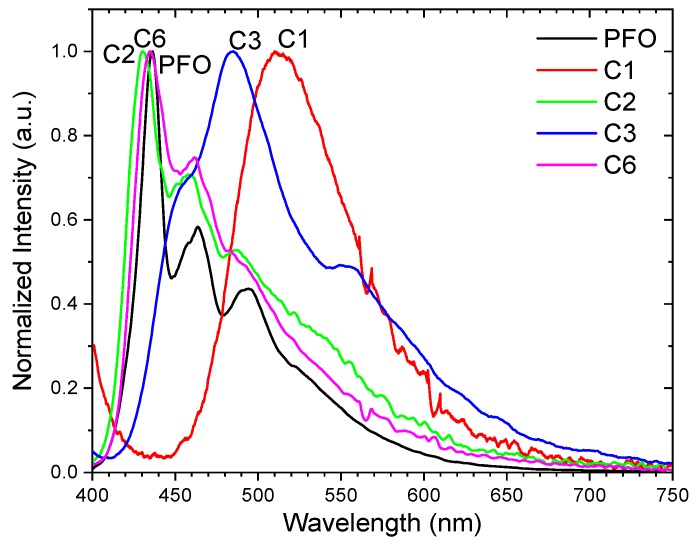
Normalized PL of the polymer films excited using a 368 nm light source.

The photoluminescence spectra of the polymer films are shown in Figure 5. C1 and C3 fluoresce in the green at ≈513 and 490 nm, while C2 and C6 emit at ≈435 nm when excited by a light source of 368 nm. Thus, polymer C1 with a pyrene side chain had the largest Stokes shift of 123 nm, followed by C3, C6, and C2. The green emission coincided with those obtained in pyrene-doped PFO structures, where the emission at ≈515 nm was directly related to the pyrene concentration of the structures [21]. Thus, the presence of pyrene in the side chain in C1 played a main role in the green emission observed in this work. It is noted that the spectra of C3 was rather broad, spanning from 400–700 nm. PL peaks of C2 and C6 were close to those of PFO, but the spectra were broadened as compared to the PL of PFO where the PL peaks lay at 435 nm, 464 nm, and 493 nm.

### 3.2. Electroluminescence Characteristics

**Figure 6:**
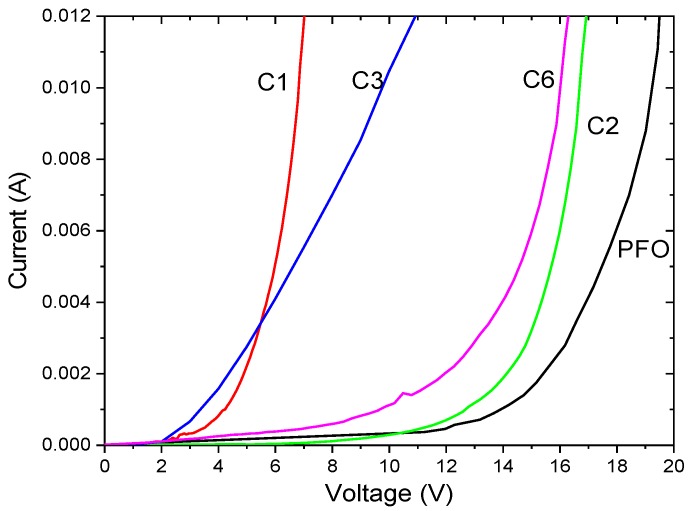
I–V curves for the single-layer device ITO/polymer/Al with PFO, C1, C2, C3, and C6.

**Figure 7:**
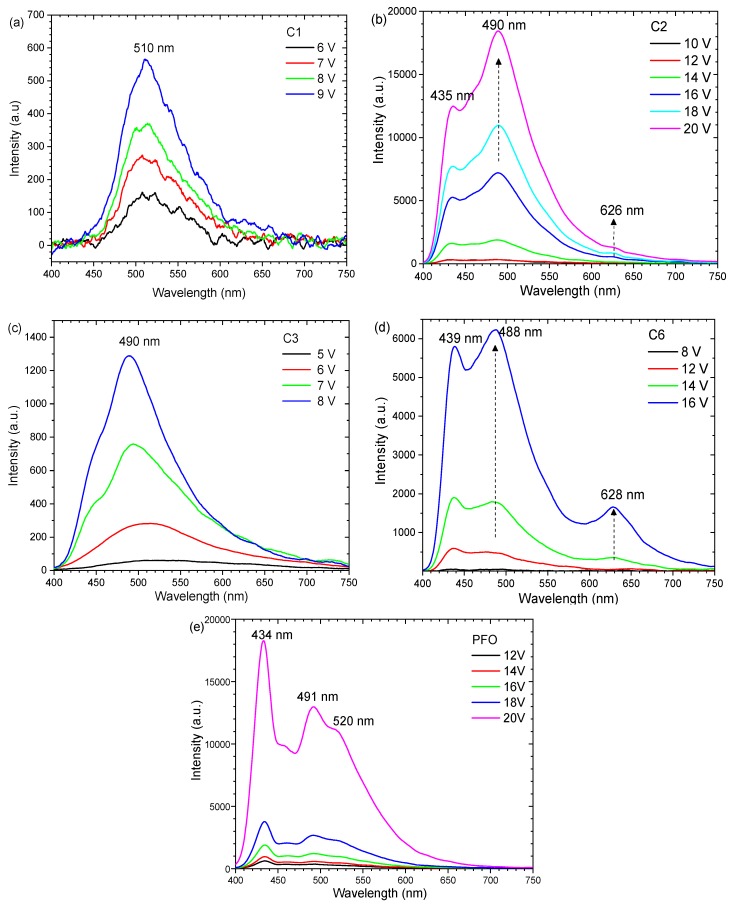
EL spectra of (**a**) C1, (**b**) C2, (**c**) C3, (**d**) C6 as compared to (**e**) PFO at different applied voltages.

All the single-layer devices produced electroluminescence. The current–voltage characteristics of the single-layer devices of C1, C2, C3, C6, and PFO are shown in Figure 6. C1 and C3 operated at a lower voltage of ≈5–10 V, while the operating voltage for C2, C6, and PFO were ≈10–20 V. The emission intensity from C1 was the lowest among all the samples due to the lower concentration and thickness of the sample in this study. C2, C6, and PFO also exhibited higher current injections as compared to C1 and C3. The EL spectra of the devices are shown in Figure 7. The EL spectra of C1 and C3 consisted of a broad peak centered at ≈510 nm and 490 nm, respectively, close to their PL spectra. The EL spectra from C3 spanned across the visible region from 400–700 nm.

Two emissions peaks were observed for both C2 and C6 at 430 nm and 495 nm, while an additional peak at 520 nm was detected for PFO. The peak at 520 nm at the green emission band may be caused by oxidation effects [17]. In the report, the effects were stabilized by the presence of TPA in the polymer. The overall spectra width was broader than the PL spectra for these polymers. The intensity of PFO increased with applied voltage consistently without significant change in the spectral shape. However, for C2 and C6, the increase of the applied voltage induced changes to the spectral shape. For C2, the ratio of the intensity at 435 nm to 495 nm increased from when the voltage was increased from 10 V to 20 V. Another peak at 626 nm emerged when the applied voltage was 18 V. For C6, the ratio of the peak at 439 nm to 488 nm changed from 1:0.8 to 1:1.1, and another peak at 628 nm occurred above 14 V. The devices of C2 and C6 appeared blue-white at higher voltage. The detailed spectral characteristics of each polymer are plotted in Figure S5.

### 3.3. Discussion

**Figure 8:**
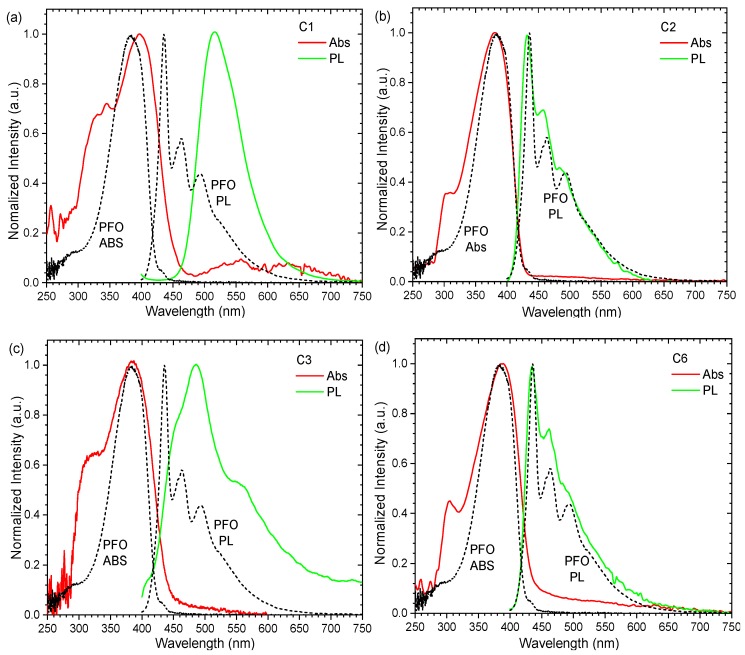
Absorbance and PL of the polymer films (**a**) C1, (**b**) C2, (**c**) C3, and (**d**) C6 as compared to a PFO film (black dotted line).

In order to investigate the emission spectral characteristics of the polymers, the absorbance and emission spectra of the polymers were compared to those of a PFO, as seen in Figure 8. For all the polymers, the main absorption peak of the polymers remained within 380–390 nm, unlike those that have been obtained with TPA connected through a vinylene bridge to PFO, which absorbed at ≈360 nm [16]. However, as a result of peak shifting and broadening, the following observation was recorded. The spectral overlapping of the absorption and PL peak for C1 and C3 caused Forster energy transfer where PL peaks at ≈430 nm and 450 nm from PFO structures were absent (Figure 8a,c). The absorbance peak of C1 broadened and shifted, covering up to ~480 nm, thus absorbing emissions <480 nm from the PFO structure. This resulted in a large Stokes shift, and PL occurred only at 515 nm. The absorbance peak of C3 broadened to ≈450 nm where emission at <450 nm would be re-absorbed and left with some residue emission at 450 nm. This resulted in a PL peak at 490 nm. The same effects were observed in another report where PFO was blended with PFM (with PFO and TPA in the backbone) [23]. In the report, deep blue emission was converted into a longer wavelength as the amount of PFM increased. C2 and C6 were less affected by the energy transfer because of less overlapping (Figure 8b,d). The higher amount of overlapping between the absorption of the polymer and the emission of a PFO structure led to a larger red shift in the order of pyrene (C1) > naphthalene (C3) > isopropyl (C6) > diphenyl (C2). The larger side chain of pyrene (C1) and naphthalene (C3) induced broadening of the absorption spectra and resulted in an emission toward the green region.

At higher operation voltage, devices of a single polymer of C2 or C6 exhibited interesting EL with multiple peaks that spanned from the blue to red regions. The emission at ≈435 nm were assigned to the excitonic emission, while the peak at 490 nm could be due to the excimers based on the reported polyfluorene derivatives [24]. At high applied voltage, the EL spectra were different from the PL spectra due to the complex charge transport and recombination that could be involved (Figure S5). The distinct difference in PL and EL peaks has been observed in some polymers with PFO and/or TPA structures. A large red-shift in the EL peak was detected for polymers with a TPA structure (PBPITP) [25]. The PL peak was detected at ≈470 nm, but EL occurred at 618 nm, indicating a different recombination mechanism of charge carriers for PL and EL. In another report, polymers with PFO in the backbone and a TPA side chain were studied [17]. The TPA side chain was able to stabilize the blue emission from the polymer. However, a small EL peak at 630–650 nm was detected in the high voltage range. The origin of the additional peak in the EL spectra can be contributed by a few mechanisms. First, a keto defect can form as a result of photooxidation or thermal oxidation. However, it normally induced an additional peak at 2.2–2.3 eV (539–564 nm) for PF [26]. More importantly, TPA is electroactive where it can be excited by high applied voltage [27]. A TPA electromer or excited-state complex could be formed between molecules or subunits carrying a charge [22]. In our previous report, both C2 and C3 were shown to have fully reversible electrochromic properties [15,20]. C2 switched to green and blue, which may account for the increase of the 490 nm peak in Figure 7b. However, the red EL peak from an OLED has not been observed before. The characteristics may be related to electron removal at the N atom from the TPA unit in the main chain, which was observed in a polymer with only a PFO-TPA main chain (referred to as M2 in the report) [28]. When tested for its electrochromic properties, PFO-TPA thin film switches from a transmissive neutral state (pale yellow) to a fully oxidized state (red). We performed a measurement on the electrochromic properties of C6, the results indicated a colour transition to red upon an applied voltage of 1.2 V. Finally, the red emission may also be due to a delayed fluorescence process. The occurrence of a delayed fluorescence has been observed in amino endcapped poly[9,9-bis(2-ethylhexy1-8-luorine-2,7-diyl] where the amino consisted of TPA units. Low temperature PL measurements at 15 K revealed that the red emission occurred as a delayed fluorescence from triplet excitons [29]. Delayed fluorescence or phosphorescence is always red-shifted to occur at a longer wavelength. Optical excitation produce singlet excited states, while some triplet states can only be formed via intersystem crossing from the singlet excited state at room temperature. Thus, triplet luminescence can hardly be detected in PL measurements at room temperature. However, in electrical excitation, charge injection occurred to form either singlet or triplet states in addition to the intersystem crossing from singlet to triplet states [30]. Thus, the overall probability of radiative recombination to produce phosphorescence is increased in electrical excitation. Such a process requires sufficiently high carrier injection at higher operating voltage.

**Figure 9:**
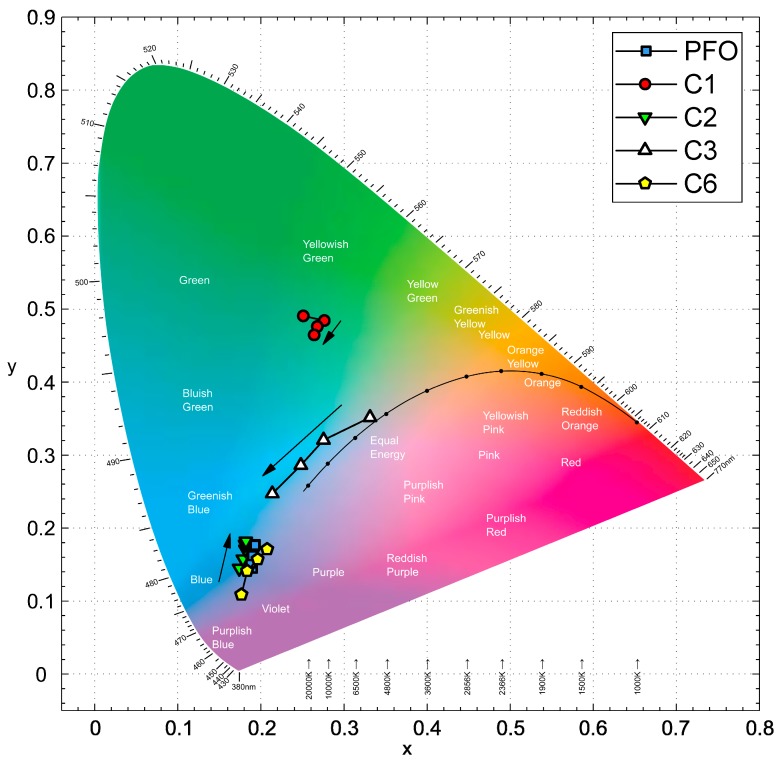
Chromaticity of C1, C2, C3, C6, and PFO devices. The increase of applied voltage (indicated by the black arrow) shifts the emission region.

The EL spectra of each polymer is converted to the CIE coordinate and overlaid onto the approximate color regions on a CIE 1931 x,y chromaticity diagram (Figure 9). The emission from C1 lay within the green region, while C3 emitted at almost the center of the chart, and there was a shift as the voltage increased. The emissions from C2 and C6 were at the edge of the blue-violet region. C2 emitted in the greenish-blue region, but as the voltage increased, the resultant emission shifted when the intensity of the 490 nm peak increased. For the devices based on C6, the emissions in the blue-shifted towards the center region with increasing voltage at high concentration, largely due to the appearance of red emissions at 628 nm.

## 4. Conclusions

A PF derivative with TPA exhibited interesting electroluminescence properties. Polymers with the same back bone of poly[(9,9-dioctylfluorenyl-2,7-diyl)-co-triphenylamine] with different side chains were synthesized, and their PL and EL as a single layer OLED were obtained. The different side chains greatly influenced the electroluminescence of the polymers. The polymers with a larger sidechain of pyrene (C1) and naphthalene structure (C3) suppressed the blue emission for PFO because of Forster energy transfer and resulted in a green emission. Polymer C2 and C6 exhibited multiple EL peaks at ≈435 nm, 490 nm, and 625 nm. The ratios of the peaks were dependent on the applied voltage. The results suggest tunability of the emission by varying the applied voltage. The occurrence of the red peak at ≈625 nm observed in EL but not PL spectra suggest that the recombination mechanisms are complex. The origin of the peak may be related to the electroactive nature of TPA units or from emission from triplet states, which are dependent on the applied electric field.

## Figures and Tables

**Table 1:** Electrochemical Properties of the Conjugated Polymers C1, C2, C3, and C6.

Polymer	HOMO *^a^* (eV)	LUMO *^a^* (eV)	E_g_ *^b^* (eV)
C1*^c^*	−4.96	−2.20	2.76
C2*^c^*	−5.03	−2.19	2.84
C3*^c^*	−4.86	−2.08	2.78
C6	−5.16	−2.26	2.90

*^a^* Calculated using the equations: HOMO = −($E_{\mathrm{onset}}^{\mathrm{ox}}-E_{\mathrm{onset}}^{\mathrm{Fc}}$) − 4.8 and LUMO = HOMO + band gap. *^b^* Calculated from the UV absorption spectrum of the polymer films using the equation: band gap (eV) = 1240/$\lambda_{\mathrm{onset}}^{\mathrm{abs}}$. *^c^* Data of C2 derived from our previous work [15], and C1, C3 from references [19,20].

## Supplementary Materials

The following are available online at https://www.mdpi.com/2073-4360/11/5/840/s1: Figure S1. Structure of M6 monomer, Figure S2. Structure of conjugate polymer C6, Figure S3. NMR Spectra of C6 (**a**) 1H NMR (**b**) 13C NMR, Figure S4. Cyclic voltammogram of the conjugated polymer C6 films on ITO-coated glass in acetonitrile solutions containing 0.1 M TBAP under argon atmosphere; the scan rate was 100 mV/s. $E_{\mathrm{onset}}^{\mathrm{ox}}$ of C6 were 0.76V via calculation. The HOMO for C6 was ≈5.16 eV. The HOMO of C6 was calculated using the empirical formula HOMO = −($E_{\mathrm{onset}}^{\mathrm{ox}}-E_{\mathrm{onset}}^{\mathrm{Fc}}$) − 4.8, Figure S5. Absorbance, PL, and EL spectra of (**a**) C1, (**b**) C2, (**c**) C3, (**d**) C6, and (**e**) PFO.
