# Supplementary material for: Photophysical and Electroluminescence Characteristics of Polyfluorene Derivatives with Triphenylamine
Source: Polymers (Basel). 2019 May 9;11(5):840. doi: 10.3390/polym11050840 (PMC6571905; doi:10.3390/polym11050840)
Supplement: Supplementary file 1 [file polymers-11-00840-s001.pdf]

Supplementary Information

Photophysical and electroluminescence characteristics of polyfluorene derivatives with triphenylamine

Qiang Zhang[1], Po-I Wang[1], Guang Liang Ong[2], Shen Hoong Tan[2], Zhong Wei Tan[2], Yew Han Hii[2], Yee Lin Wong[2], Khee Sang Cheah[2], Seong Ling Yap[3], Teng Sian Ong[2], Teck Yong Tou[2], Chen Hon Nee[2*], Der Jang Liaw[1**], and Seong Shan Yap[2]

[1]Department of Chemical Engineering, National Taiwan University of Science and Technology, 10607 Taipei, Taiwan

[2]Faculty of Engineering, Multimedia University, Jalan Multimedia, Cyberjaya 63100 Malaysia

[3]Department of Physics, University of Malaya, Lembah Pantai, 50603 Kuala Lumpur, Malaysia

Corresponding authors: *neechenhon@gmail.com; **liawdj@gmail.com

Material

The monomer M1, M2, M3 and conjugate polymer C1, C2, C3 were synthesized based on our previous work.[1-3] *N*-(4-aminophenyl)-*N*-phenyl-1-aminopyrene was prepared according to reported procedure.[4] 1-Bromo4-iodobenzene, bis(dibenzylideneacetone)palladium (Pd(dba)$_2$), 1,1'-bis(diphenylphosphino)ferrocene (DPPF), 4-Isopropylaniline, sodium *tert*-butoxide and tetrakis(triphenylphosphine)palladium(0) (Pd(PPh$_3$)$_4$) were purchased from Acros Organics. Anhydrous potassium carbonate (K_2CO_3) was purchased from Fisher Chemical. 9,9-Dioctylfluorene2,7-diboronic acid bis(1,3-propanediol) ester was purchased from Sigma-Aldrich Chemical. Tetrabutylammonium perchlorate (TBAP) was obtained from TCI and recrystallized from ethyl acetate twice before being dried in a vacuum prior to use. The solvents (analytical grade) were purchased from Merck. Tetrahydrofuran and toluene were distilled from sodium/benzophenone (deep purple) under nitrogen before use. All other reagents were used as received.

Synthesis of *N,N*-bis(4-bromophenyl)- l,4-phenylenediamine (M6)

1 equiv of 4-isopropylaniline, 2 equiv of 1-bromo4-iodobenzene, 0.02 equiv of $Pd(dba)_2$, 0.02 equiv of 1,10-bis(diphenylphosphino)ferrocene, 2 equiv of sodium tert-butoxide, and dry toluene were charged in a three-necked flask and kept under nitrogen atmosphere. The mixture was heated and stirred to reflux for 6 h. After the completion of the reaction, the solvent was removed under reduced pressure, and the residue was extracted with dichloromethane/ water. The collected organic layer was dried over $MgSO_4$ overnight and then filtered to remove $MgSO_4$. The solvent from the filtrate was then removed using vacuum distillation, and the residue was purified by silica gel column chromatography (dichloromethane : n-hexane = 1:3) to obtain products. (Yield : 85 %). ^{1}H NMR (600 MHz, $CDCl_3$, Me_4Si): δ (ppm) = 1.27 (6H, H_1) , 2.89 (1H, H_2) , 6.94 (4H, H_5) , 7.00 (2H, H_4) , 7.14 (2H, H_3), 7.33 (4H, H_6). ^{13}C NMR (150 MHz, $CDCl_3$, Me_4Si): δ (ppm) = 146.7 (C_{10}), 144.73 (C_7), 144.42 (C_8), 132.21 (C_6), 127.49 (C_3), 125.01 (C_5), 124.92 (C_4), 114.98 (C_9), 33.51 (C_2), 23.98 (C_1).

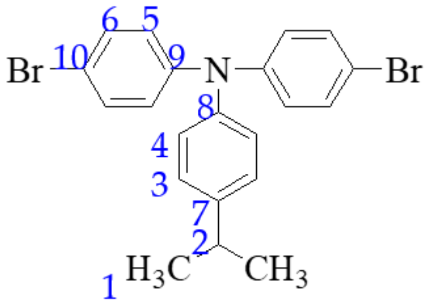

Figure S1. Structure of M6 monomer

General procedure of Suzuki coupling polymerization

The 1 equiv of dibromo monomers, 1 equiv of diboronic ester monomer and 0.02 equiv of $Pd(PPh_3)_4$ were dissolved in degassed toluene in a three-neck round-bottom flask with a condenser. The 2 M aqueous solution of K_2CO_3 was added to the mixture. The resulting solution was stirred at 105 °C for 48 h under the flow of nitrogen. After cooling, the solution was extracted with toluene/water for three times. The organic layer was separated, concentrated by rotary evaporator and precipitated in excess methanol. The products was filtrated and further purified by Soxhlet extraction with acetone for 48 h.

Synthesis of conjugate polymer (C6)

C6 was synthesized according to the procedure described above. (Yield : 81 %). The structure of C6 (Fig. S2) is verfied by NMR (Fig. S3). The cyclic voltammograms of C6 is shown in Fig. S4.

^{1}H NMR (600 MHz, $CDCl_3$, Me_4Si): δ (ppm) = 0.65-0.85 (20H, H_{19}, H_{25}), 1.07-1.20 (20H, H_{20}, H_{21}, H_{22}, H_{23}, H_{24}), 1.28 (6H, H_1), 2.02 (4H, H_{18}), 2.92 (m, 2H, H_2), 7.13-7.95 (4H, H_3, H_4), 7.23 (d, 4H, H_5), 7.54-7.62 (8H, H_6, H_{12}, H_{15}), 7.73-7.76 (2H, H_{13}). ^{13}C NMR (150 MHz, $CDCl_3$, Me_4Si): δ (ppm) = 14.05 (C_{25}), 22.59 (C_{24}), 23.83 (C_{19}), 24.07 (C_1), 29.21 (C_{22}, C_{23}), 30.05 (C_{21}), 31.78 (C_{20}), 33.56 (C_2), 40.49 (C_{18}), 55.21 (C_{17}), 119.88 (C_{13}), 120.92 (C_{15}), 123.82 (C_5), 124.96 (C_3), 125.49 (C_{12}), 127.24 (C_4), 127.75 (C_6), 135.50 (C_{10}), 139.39 (C_{11}), 139.74 (C_{14}), 144.15 (C_8), 145.10 (C_7), 147.05 (C_9), 151.64 (C_{16}).

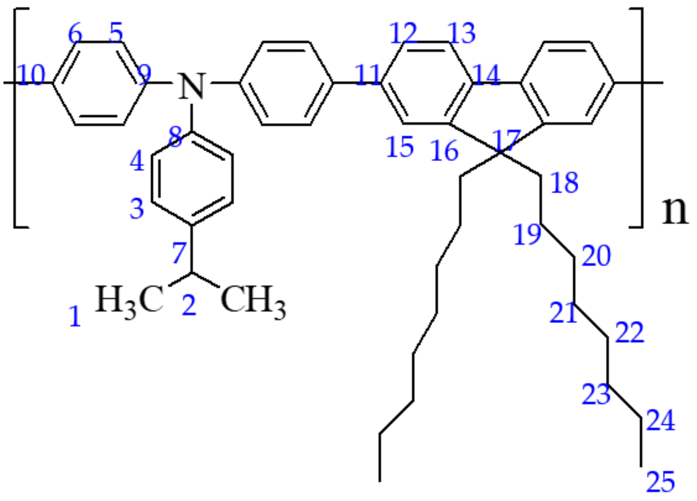

Figure S2. Structure of conjugate polymer C6

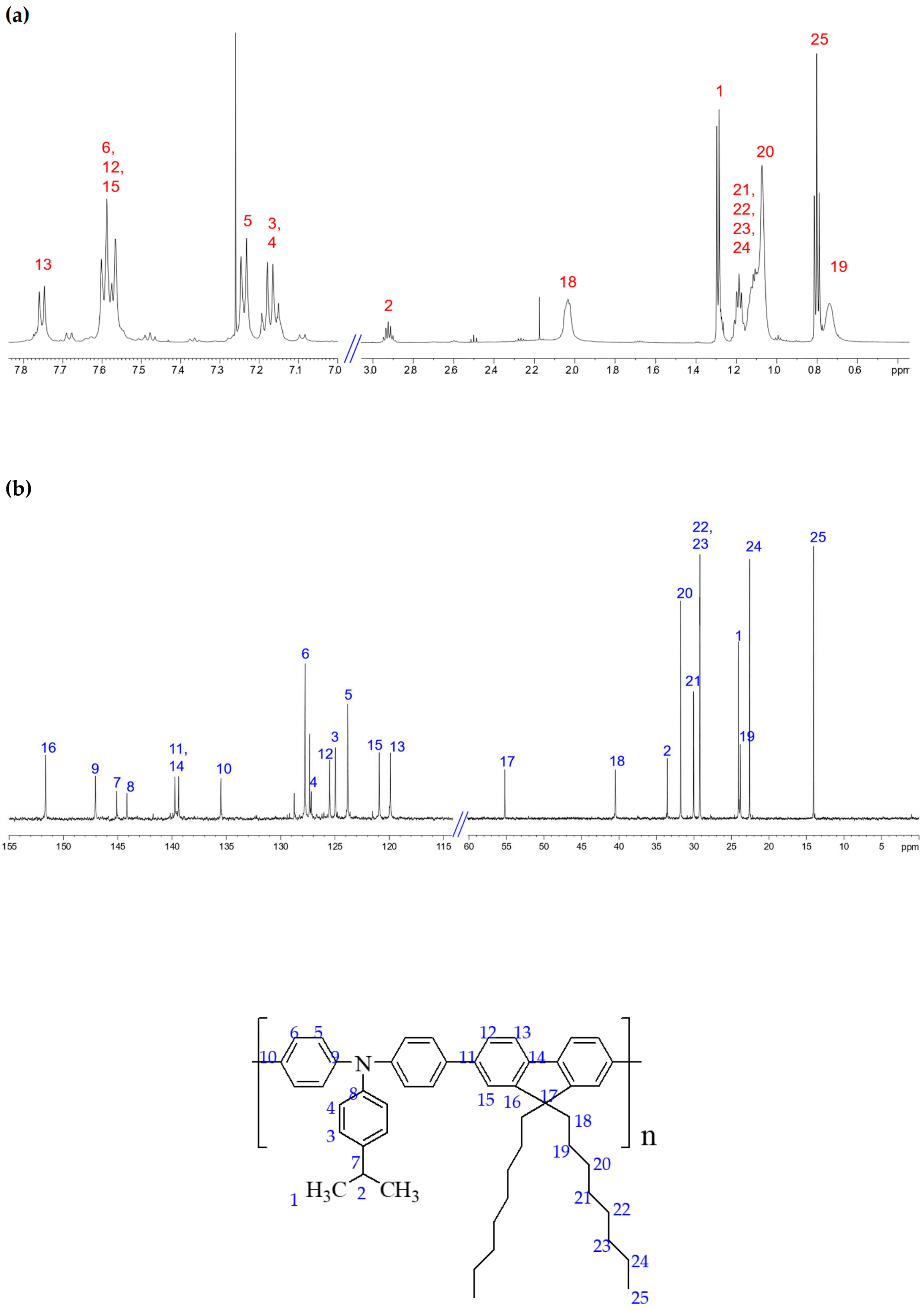

Figure S3. NMR Spectra of C6 (a) ^{1}H NMR (b) ^{13}C NMR

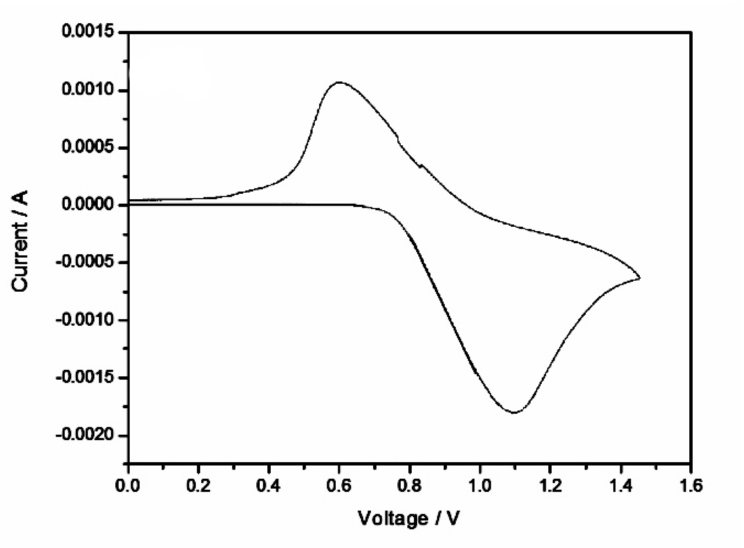

Figure S4. Cyclic voltammograms of the conjugated polymer C6 films on ITO-coated glass in acetonitrile solutions containing 0.1 M TBAP under argon atmosphere; the scan rate was 100 mV/s. E^{ox}_{onset} of C6 were 0.76V, by calculation the HOMO for C6 were ~5.16 eV. The HOMO of C6 is calculated by the empirical formula HOMO = $-(E^{ox}_{onset} - E^{Fc}_{onset}) - 4.8$.

The absorbance, PL and EL spectra of the polymers

The absorbance, PL and EL spectra of C1, C2, C3, C6 and PFO are shown in Fig. S5.

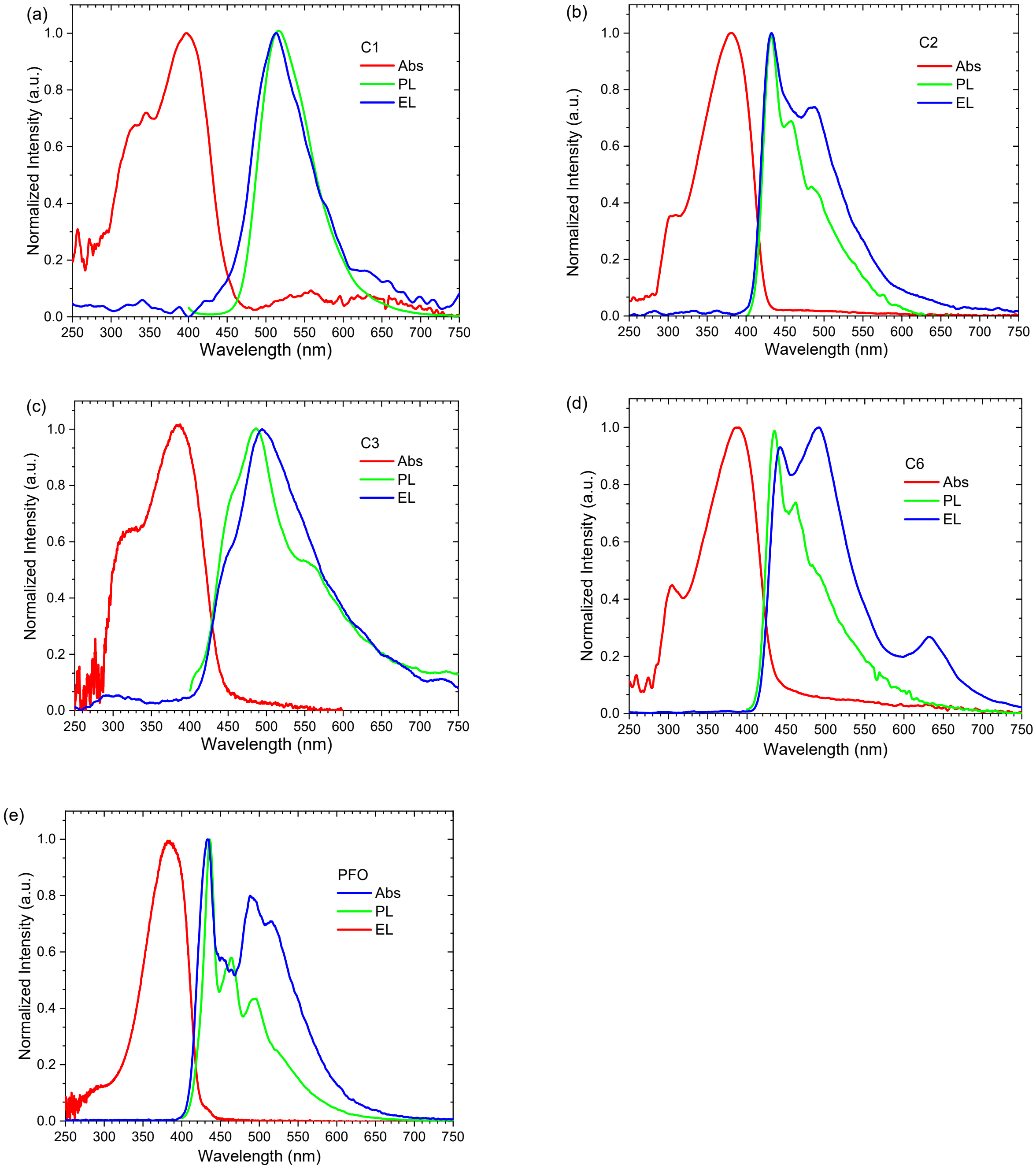

Figure S5. Absorbance, PL and EL spectra of (a) C1, (b) C2, (c) C3, (d) C6 and (e) PFO.

Reference

1. Zhang Q, Tsai CY, Abidin T, Jiang JC, Shie WR, Li LJ, et al. Transmissive-to-black fast electrochromic switching from a long conjugated pendant group and a highly dispersed polymer/SWNT. Polym. Chem. 2018;9:619–26.

2. Chen W, Wang K, Hung W, Jiang J, Liaw D-J, Lee K-R, et al. Novel triarylamine-based alternating conjugated polymer with high hole mobility: Synthesis, electro-optical, and electronic properties. J. Polym. Sci. Part A Polym. Chem. [Internet]. Wiley Subscription Services, Inc., A Wiley Company; 2010 Nov 1;48:4654–67.

3. Wu HY, Wang KL, Liaw DJ, Lee KR, Lai JY. Electrochromic material containing unsymmetrical substituted N,N,N0,N0-Tetraaryl-1,4-phenylenediamine: Synthesis and their optical, electrochemical, and electrochromic properties. J. Polym. Sci. Part A Polym. Chem. [Internet]. Wiley Subscription Services, Inc., A Wiley Company; 2010 Apr 1 [cited 2017 Sep 26];48:1469–76.

4. Lai YC, Kurosawa T, Higashihara T, Ueda M, Chen WC. Donor-Acceptor Oligoimides for Application in High-Performance Electrical Memory Devices. Chemistry - An Asian Journal 2013, 8, 1514–1522. Chem. Asian J. 2013;8:1514–1522.
